# A Cross-Sectional Study on Hyomental Distance Ratio (HMDR) as a New Predictor of Difficult Laryngoscopy in ICU Patients

**DOI:** 10.7759/cureus.25435

**Published:** 2022-05-28

**Authors:** Dr Hrithma, Rooparani K, Dr Thejeswini Mahadevaiah, Vikas K N

**Affiliations:** 1 Department of Anaesthesiology, MS Ramaiah Medical College, Bengaluru, IND

**Keywords:** difficult airway, icu, difficult intubation, laryngoscopy, hyomental distance ratio

## Abstract

Background: Intubation in the ICU is sometimes unpredictable unlike in an operation theatre, where pre-anesthetic assessment for airway has been done. This study has been done to evaluate the usefulness of hyomental distance ratio (HMDR) in accurately predicting difficult laryngoscopy in ICU patients.

Methods: In this study, 104 critically ill patients in the age group 18-70 years, undergoing tracheal intubation in ICU were included. A hard plastic ruler was pressed on the skin surface just above the hyoid bone and the distance to the tip of the anterior-most part of the mentum measured was defined as hyomental distance (HMD). HMD was assessed in neutral and extended head positions, and the HMDR was calculated. All patients were sedated, pre-oxygenated, induced, and relaxed prior to glottic visualization by direct laryngoscopy, which was performed by an experienced anesthetist. Cormack-Lehane's (CL's) grade was assessed without external laryngeal manipulation. Further management was as per ICU protocol.

Results: Using the Chi-Square test for statistical analysis, a p-value of HMDR in assessing difficult laryngoscopy was found to be <0.001 suggesting strong significance. HMD in the extended head position (HDMe) showed moderate significance with a p-value of 0.047. HMDR <1.2 can be considered a clinically reliable individual predictor of difficult laryngoscopy in ICU patients.

Conclusion: HMDR <1.2 can be used as a simple, easy, and reliable bedside test to predict difficult laryngoscopy amongst ICU patients. An optimal combination of tests is suggested if feasible for better accuracy.

## Introduction

Endotracheal intubation in places like intensive care unit (ICU) is most of the time associated with risks and differs from intubation in operating theater for various reasons. Patients coming for elective surgery are well optimized prior to intubation, in contrast to critically ill patients who may have poor physiologic reserve and mostly require emergency intubation. Therefore, there is limited time for the optimization of parameters [1-4]. Complications that are associated with emergency intubations in critical patients are more than 20% [3-6]. However, evaluation of the airway prior to intubation depends on multiple clinical parameters with varying predictions for difficult airways. 

Hyomental distance ratio (HMDR) is one such simple parameter that is easy to identify clinically, can be applied bedside readily, and is clear to use. Hyomental distance (HMD) has been used to estimate mandibular space, and has shown only modest degree of accuracy in diagnosis [7]. SSuyama found that HMD <3.0 cm was a good predictor of the difficulty in visualization of the larynx (DVL) during direct laryngoscopy. He concluded that mallampati is the best predictor [8]. Takenaka et al., in their study, demonstrated that HMDR accurately predicted occipito-atlanto-axial (OAA) complex extension capacity [9]. Huh concluded that the HMDR had a threshold of 1.2 for clinical prediction of DVL [10]. Kazelic et al. have observed that HMDR <1.2 is a valuable predictor of DVL to be performed on regular basis [11].

All the above-mentioned studies were performed in an operating theater where patients are usually hemodynamically stable with normal airway anatomy. There are limited studies performed on ICU patients. This study is intended to evaluate the usefulness of HMDR in the prediction of difficult laryngoscopy for ICU patients.

## Materials and methods

A cross-sectional double-blinded study was performed on the patients of both genders, with ages between 18-70 years, requiring intubation in ICU from December 2019 to November 2021 in MS Ramaiah Hospital, MSR Nagar, Bengaluru. After ethics committee clearance (MSRMC/EC/AP-63/10-2019), written and informed consent was obtained from all the patients before enrolling in the study. Pregnant patients, patients with midline neck swellings, cervical injury, mouth opening <3 cm, recent head and neck surgery, and upper airway disease (maxillary/facial fracture) were excluded.

Each patient was made to undergo a pre-anesthetic assessment of the airway. The patient was positioned supine with their head firmly placed on the ICU bed. The head was kept in a neutral position by closing the mouth. A hard-plastic ruler was used, which was pressed over the hyoid bone tip and distance between the tip and anterior of the mentum was recorded and it was defined as HMD taken at the neutral head position (HMDn). The latter head was extended to the maximum taking care shoulders should not be lifted during the extension of the head. HMD, which was again measured in this position, was defined as HMD in the extended head position (HMDe). The HMDR was derived as the ratio of HMDe to HMDn. 

All patients were initially pre-oxygenated and later sedated using midazolam 0.5 mg and fentanyl 2 mcg/kg. The patient's head was positioned using the head ring to get a sniffing position. Patients were all induced by using intravenous (IV) agent propofol (1.5 mg/kg) and paralyzed using succinylcholine (1.5 mg/kg), which enabled good oro-tracheal intubation.

Laryngoscopy was done by an anesthetist with intubation experience of two years who was blinded to the HMDR value of the patient, with the help of an appropriate McIntosh blade. Glottis visualization was graded as per Cormack-Lehane (CL) grading, without manipulation of the larynx externally [12-17]. However, laryngeal pressure was later permitted externally after evaluating CL grading for insertion of the endotracheal tube. CL grades 3 and 4 were defined as DVL. The study ended once the CL grade was assessed. Further airway management of patients was performed as per ICU protocol and patient requirements.

Statistical analysis

Kazelic et al. had observed that the sensitivity of detecting difficult intubation (DI) with HMDR <1.2 was 95.6%. In our study, to expect a similar result with a 95% confidence interval and 4% precision, a required minimum of 101 subjects was estimated [11]. Keeping in view of the drop-outs, we decided to keep a total of 104 patients in our study. 

Inferential and descriptive statistical analyses were done in our study using the statistical software SPSS 22.0. Results for continuous measurements were taken as mean ± SD (min to max) and categorical measurements were taken as number (%). The level of significance was taken at 5% or p-value <0.05. Chi-square or FisherExact test was utilized to get significance in the parameters on the categorical scale among two/more groups, Non-parametric setting was taken for qualitative data analysis. FisherExact test was taken whenever the cell samples were found to be too small. Student t-test was utilized in finding significance in parameters on a continuous scale among two groups on metric parameters. Leven's test was performed for assessing homogeneity of variance.

## Results

**Figure 1:**
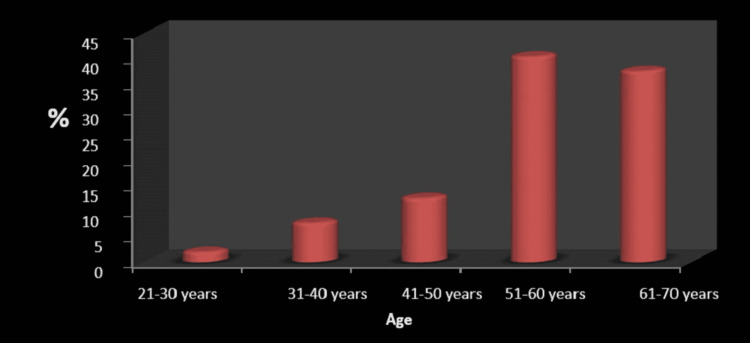
Age in years—frequency distribution of patients

A total of 104 patients were studied. 72 of them were male (69.2%) and 32 were female (30.8%). Male preponderance was noted in this study (Table 1). The average age of the subjects included in this study was around 56.68 ± 9.60 (Figure 1). Subjects with CL grades 3 and 4 were classified as having difficulty in laryngoscopy, which was noted in 26 out of 104 subjects studied.

**Table 1:** Gender—frequency distribution of patients studied

Gender	No. of Patients	%
Male	72	69.2
Female	32	30.8
Total	104	100.0

HMDe vs DVL

The DI prediction by HMDe and actual DVL were compared. 10 of 26 patients with HMDe ≤5.3 cm had DVL, while 16 of 79 patients with HMDe >5.3 cm had DVL (Table 2). The sensitivity of HMDe for predicting difficulty in laryngoscopy was 38.5% and the specificity found was 80.8%. The test had a positive predictive value (PPV) of 40%, and a negative predictive value (NPV) of 79.8% (Table 3). 

**Table 2:** Association of HMD in relation to difficult laryngoscopy *p<0.05

Hyomental Distance (HMD)	Difficult Laryngoscopy		Total	P Value
	Absent	Present		
HMD in the extended head position (HMDe)				
•≤5.3	15 (19.2%)	10 (38.5%)	25 (24%)	0.047*
•>5.3	63 (80.8%)	16 (61.5%)	79 (76%)	
HMD in the neutral head position (HMDn)				
•≤5.5	69 (88.5%)	21 (80.8%)	90 (86.5%)	0.320
•>5.5	9 (11.5%)	5 (19.2%)	14 (13.5%)	
Hyomental distance ratio (HMDR)				
•≤1.2	11 (14.1%)	23 (88.5%)	34 (32.7%)	<0.001**
•>1.2	67 (85.9%)	3 (11.5%)	70 (67.3%)	
Total	78 (100%)	26 (100%)	104 (100%)	

**Table 3:** Correlation of findings of hyomental distance (HMD) to predict difficult laryngoscopy TP: True positive, TN: True negative, FP: False positive, FN: False negative, PPV: Positive predictive value, NPV: Negative predictive value, Sp: Specificity, Se: Sensitivity * indicates statistical significance (p<0.05)

Hyomental distance (HMD)	Observation					Correlation					
	TP	FP	FN	TN	Total	Se (%)	Sp (%)	PPV (%)	NPV (%)	Accuracy	P value
HMD in the extended head position (HMDe)	10	15	16	63	104	38.5	80.8	40.0	79.8	70.2	0.011*
HMD in the neutral head position (HMDn)	21	69	5	9	104	80.8	11.5	23.3	64.2	28.8	0.148
Hyomental distance ratio (HMDR)	23	11	3	67	104	88.5	85.9	67.7	95.7	86.5	<0.001*

HMDn vs DVL

Prediction of intubation difficulty by HMDn and actual DVL were compared. Five out of 14 patients with HMDn >5.5 cm had DVL, while 21 out of 90 patients with HMDn <5.5 cm had DVL (Table 2). The sensitivity for HMDn in predicting DVL was 80.8% and the specificity found was 80.8%. This test had a PPV of 23.3% and NPV of 64.2% (Table 3).

HMDR vs DVL

Prediction of intubation difficulty by HMDR and actual DVL were compared. 23 of 34 patients with that HMDR ≤1.2 had DVL, while three of 70 patients with HMDR >1.2 had DVL (Table 2). The sensitivity of HMDR in predicting DVL was measured to be 88.5% and the specificity of 85.9%. The test had a PPV of 95.7%, and NPV of 86.5% (Table 3).

## Discussion

DI is generally correlated to DVL. Therefore, early recognition of patients who are at risk for DVL is vital in following safe and specialized alternative approaches for intubation [12]. It is essential to identify simple, easy, and bedside clinical parameters that can accurately predict difficult laryngoscopy [13]. Various studies have indicated the diagnostic utility of HMDR and other parameters in predicting intubation in patients posted for elective surgeries. The term 'HMDR' was coined by Takenaka et al. [9]. 40 patients with rheumatoid arthritis were studied. HMDR predicted reduced OAA extension capacity. However, there are lesser studies evaluating the reliability of HMDR as a predictor of DVL in ICU patients. Consequently, this study was performed to assess validity diagnostically for HMDR in DVL in ICU patients requiring tracheal intubation admitted to MS Ramaiah Teaching Hospital from December 2019 to November 2021. The airway predictors HMDn, HMDe, and HMDR were examined. 

Demographics

A total of 104 patients aged >18 years of age, admitted in ICU requiring intubation were taken for this study. The majority of them were male 72 (69.2%) and the remaining were female 32 (30.8 %) with an overall mean age of 41.8 years. Our study showed that DVL was present more among males than females (27.8% vs 18.8 %), this data was not statistically relevant as the majority of study subjects included were also male.

Incidence

Visualizing the larynx is mostly described by CL grades with grades 3 and 4 labeled as DVL. The incidence of DVL in various studies is 1.5% to 8% among general surgery patients it is more with cervical spine (20%)/laryngeal surgery (30%). In this study, the DVL found was in 26 (25%) out of 104 patients studied, which is almost similar to previous studies. None of the intubations failed. In one meta-analysis of 14,438 patients, the incidence of DVL was 6%-27% [14]. Huh et al. studied 213 apparently normal patients who underwent general anesthesia with intubation and reported a 12.2% incidence of DVL [10]. The disparity in the incidence of DVL may be attributed to age, ethnicity, or procedural alterations such as the laryngoscopy blade used [15,16]. 

Specificity and sensitivity of HMDR

DVL predictors should ideally have 100% specificity and 100% sensitivity, but most of the time, they are inversely proportional to each other. Cutoffs used for deriving specificity and sensitivity in this study were:

• HMDe ≤5.3 cm

• HMDn >5.5 cm

• HMDR ≤1.2 

In our study, maximum sensitivity of (88.5% ) was recorded in predicting DVL with HMDR and then HMDn (80.8 %) and the specificity in this study was found to be high with HMDR followed by HMDe. These findings are in contrast to many studies where specificity was higher for all the HMD parameters. The overall accuracy of HMDR in predicting DVL was 86.5%, this parameter was considered statistically significant with p<0.001.

The sensitivity of HMDn was more whereas the specificity of HMDe was better when compared to each other. Diagnostic accuracy of HMDe for predicting DI was found to be moderately significant whereas HMDn showed no statistically significant correlation. These results were found to be comparable to the Kalezic et al. study where HMDR stood out to be a significant predictor of DI with a sensitivity of 95.6%, which is better than our study probably due to the larger sample size [11]. HMDR was suggested as a possible predictor for DVL, and we were able to confirm its use in our study to a certain extent [17]. 

Radiological studies showed that HMD increased while extending the head due to the OAA complex as well as in sub-axial regions [16]. Hyoid bone shifts along with the cervical spine while the neck and head are moved. Because of this, HMDR individually correlates with an OAA complex capacity [10]. Along with this, HMDR is quick as well as easily done and it is discovered to have more accuracy than recordings of the OAA complex angle measured through goggles mounted goniometer [18].

During laryngoscopy, creating a nearly straight line from the mouth to the glottic opening depends entirely on the degree of extension of the OAA complex [9,18]. This angle which is needed for exposing glottis while laryngoscopy found to be as at least 12°, the correlating HMDR would be calculated to 1.25 [10]. In our study, a cut-off point of 1.2 was taken based on the literature [19]. Further, we also observed that a cut-off of HMDR (≤1.2) provided statistically significant sensitivity.

This study will be very useful in ICU where physicians come across patients requiring intubation while sleeping on the bed. ICU patients will be connected to monitoring devices, IV lines, and drains, more nurses will be needed to place these patients in a sitting posture to take measurements. Sutthiprapaporn et al. demonstrated postural variation of the hyoid bone with gravity, where it was noted that body of hyoid bone moves caudally 6.7 ± 4.4 mm in response to a change from supine to sitting upright [20]. 

Limitations

There are some limitations to this study. Though we have obtained statistically significant results with the derived sample size, by studying a higher sample size, better accuracy could have been obtained. It is recommended that more studies using HMDR to predict DI should be performed with ICU patients across the globe to make it a commonly usable parameter in the ICU. Although DVL is important in determining difficulty in intubation, it does not achieve 100% success rate with just visualization of larynx. In some patients, pressure was given on larynx externally to help laryngoscopic view so that intubation could be done easily.

## Conclusions

In our study, the highest sensitivity (88.5% ) was noted for predicting the DVL with HMDR and next was HMDn (80.8%). The specificity in this study was relatively high with HMDR followed by HMDe. These recordings are in contrast to many studies where specificity was higher for all the HMD parameters. The overall accuracy of HMDR in predicting difficulty in laryngoscopy is 86.5%, and HMDR would be considered statistically significant with p<0.001.

We conclude that in the critical care set-up, HMDR could be utilized as one of the trustworthy and accurate predictors of DVL. HMDR <1.2 could be used as test threshold. Even though HMDR is a reliable predictor, we would like to suggest a combination of other tests along with HMDR if feasible, rather than using it alone.
